# Does the Number of Substitutions Used during the Matches Affect the Recovery Status and the Physical and Technical Performance of Elite Women’s Soccer?

**DOI:** 10.3390/ijerph191811541

**Published:** 2022-09-14

**Authors:** Ronaldo Kobal, Rodrigo Aquino, Leonardo Carvalho, Adriano Serra, Rafaela Sander, Natan Gomes, Vinicius Concon, Guilherme Passos Ramos, Renato Barroso

**Affiliations:** 1 School of Physical Education, University of Campinas, Campinas 13083-851, Brazil; 2 Sport Club Corinthians Paulista, São Paulo 03087-000, Brazil; 3 LabSport, Department of Sports, Centre of Physical Education and Sports, Federal University of Espírito Santo, Vitória 29075-910, Brazil; 4 Brazilian Football Confederation, Rio de Janeiro 22775-055, Brazil; 5 Laboratory of Endocrinology and Metabolism, Federal University of Minas Gerais, Belo Horizonte 31270-901, Brazil

**Keywords:** contextual factor, football, team sports, female players

## Abstract

The aim of this study was to compare the effect of a new rule for substitutions (four and five) with the rule before the COVID-19 pandemic (up to three) on recovery status, physical and technical performance, internal workload, and recovery process in elite women soccer players. Thirty-eight matches from 2019 to 2020 from the Brazilian Championships were analyzed. All data for the two conditions (≤3 and 4–5 substitutions) were compared using an independent *t*-test. The physical demands measured by a global positioning system (GPS) and the technical (obtained from Instat) and internal workload (rating of perceived exertion [RPE]) were assessed. The recovery process was measured by the total quality recovery (TQR) 24 h after each match. No differences were observed in any physical and technical parameters between 4–5 and ≤3 substitutions (*p* > 0.05). Moreover, 4–5 substitutions demonstrated lower RPE (*p* < 0.001) and workload-RPE (*p* < 0.001), higher TQR (*p* = 0.008), and lower time played by the player (*p* < 0.001), compared to ≤3. Thus, the new provisory rule for substitutions improved the balance between stress and recovery.

## 1. Introduction

Soccer is an intermittent and high-intensity team sport, which depends on endurance, strength, power, and speed abilities [1,2,3]. Elite women soccer players cover ~10 km, with ~1500 m in high intensity running, and ~200 m in sprint distance during a soccer match [4,5,6]. In addition, physical performance declines during the matches [3,7], for instance, Bradley et al. [8] observed in the women’s UEFA Champions League large decrements in the second half compared to the first half in the distance covered at speeds above 12 km·h^−1^ (1651 vs. 1500 m in the first and second half, respectively). Additionally, Mohr et al. [3] reported that the total distance covered in the first half was longer than the second half (5.28 vs. 5.00 km), and the same trend was also observed for the high intensity running (0.91 vs. 0.70 km), and sprint distance (0.25 vs. 0.21 km). Interestingly, these impairments in physical performance were not observed in the indicators of technical performance, such as successful passes, lost balls, and duels won. Thus, declines in physical performance of soccer players do not seem to affect the technical performance.

Coaches are allowed to use three substitutions per match in an attempt to create a novel team tactical strategy, to replace a player who has become injured, to maintain the physical performance during the match, and to minimize performance decrements in the second half. Bradley et al. [9] compared the physical demand covered during the soccer matches among players who completed the entire match, replaced players (who were substituted), and substituted players (who entered during the match) in the English Premier League. Substitute players covered a higher total distance and high intensity running distance when normalized by the time they played (minutes per minute, m/min) than players who were replaced or completed a full match, and replaced players covered a higher distance than players that completed a full match (120, 116, and 112 m/min; and 9.0, 8.2, and 7.1 m/min, respectively) [9]. Padron-Cabo et al. [10] demonstrated a similar finding in the Spanish League during the season 2014–2015. Starting players who completed matches covered a lower total distance/minute and high intensity running distance/minute than replaced players and substitute players (106, 111, and 111 m/min and 5.1, 6.1, and 6.7 m/min, respectively), with no difference between replaced and substitute.

In general, the measure of distance/minute is used by researchers and practitioners to investigate topics that can influence match running performance [9,11,12]. However, normalized distances should be used with caution because this measure is time-played dependent and did not support decrement of physical performance. For instance, substitute players that played 10 min and covered 120 m/min covered a total distance of the 1200 m, and players who covered 112 m/min for 90 min covered 10,080 m. Thus, relative metrics (i.e., m/min) do not allow for understanding the impact of the whole team performance. Alternatively, to investigate the influence of the substitute players on the whole team performance, it is required to sum metrics from all players who participated in the match.

Recently, the *Fédération Internationale de Football Association* (FIFA) provisionally changed the rules regarding the number of substitutions, allowing up to five by team. The rationale for this change lies in the congested periods of matches that would be experienced by players returning to play following COVID-19 lockdown. The large number of matches in a shorter period of time would cause fatigue to accumulate and increase the risk of injury [13,14]. More substitutions reduce the overall player’s match time exposure due to greater players’ rotation, which diminishes external and internal load [i.e., total distance covered and rating of perceived exertion (RPE), respectively] in some of the players. In addition, it seems that there is a negative correlation between RPE and next day recovery status, suggesting that athletes who display a high perception of exertion are those that demonstrate low recovery indexes [15]. Interestingly, athletes who show higher RPE and worse recovery status were those that presented higher injury rates and were sick more frequently [16]. Thus, increasing the number of substitutions can contribute to hasten players’ recovery after matches and to safeguard the athlete’s health by avoiding illness and injury.

Nonetheless, the effect of the number of substitutions and the provisory rule is unclear, especially in women soccer players. Therefore, the purpose of this study was to compare the effect of the number of substitutions on recovery status and the physical and technical performance in elite women soccer players. We hypothesized that the higher number of substitutions would decrease physical demands and internal load and improve the recovery status of elite women soccer players, with no changes in technical performance.

## 2. Materials and Methods

### 2.1. Study Design

This retrospective study was conducted during the 2019 and 2020 seasons in the National Brazilian Championships and 38 official matches were analyzed (19 matches in each championship). In 2019, up to three substitutions per match were allowed, whereas in 2020 the rule allowed up to five substitutions. Matches were classified according to the number of substitutions performed (i.e., 0–5). Then, all matches were grouped into 2–3 (21 matches) and 4–5 substitutions (17 matches). The physical performance was measured by GPS and RPE was assessed after all matches. The recovery process was measured by a TQR questionnaire 24 h after each match. In addition, the technical performance was determined by data provided by Instat^®^ (Instat, Moscow, Russia).

### 2.2. Participants

Twenty-four professional Brazilian women soccer players (28.0 ± 4.6 years, 163.7 ± 5.2 cm, 58.8 ± 7.7 kg, and body fat 22 ± 8.7%) from the same club participated in this study. All players participated in the National Brazilian Championship, seasons 2019 and 2020 (second and first place, respectively), thus attesting their high level of performance. Six players had already participated in the National Brazilian soccer team. Goalkeepers were excluded from the study. All participants were informed about the procedures, benefits, discomforts, and possible risks of the study and signed a free and informed consent before participation. The University’s Research Ethics Committee approved the experimental protocol.

### 2.3. Total Quality Recovery (TQR)

The TQR scale (6–20) was used to provide a means to measure psychophysiological recovery [17]. The athletes answered the question “How do you feel about your recovery?” using the TQR scale, 24 h after each match. All players were previously familiarized with the scale, and the mean results were utilized for analysis.

### 2.4. Rating of Perceived Exertion (RPE)

Athletes were familiarized with the CR-10 Borg RPE scale as this was part of their training routine. RPE was assessed with the modified Borg 10-point (0–10) scale, which is widely used in both practical and research settings [18]. This method uses a simple question: “How was your match today?”. The answer was provided 30 min after the end of training sessions and official matches, by choosing a descriptor and a number from 0–10. We considered the mean of each match in the analysis. The workload-RPE was determined by multiplying each players’ playing time (minutes) in each match by the RPE, as described by Foster [18].

### 2.5. Match Running Performance

All soccer matches activity profiles were obtained via a GPS-system operating at 10 Hz (GPS-units; Playertek, Catapult Innovations, Melbourne, Australia). GPS devices were fitted to the upper back of players using a harness and the same unit was used by each player in all measures to reduce inter-unit measurement error. Units were turned on 10 min before each match. Thirty-eight official matches were measured using GPS. The activity profiles measured were total distance covered (km), total sprint distance (m) (speed > 18 km·h^−1^), number of accelerations and decelerations (>3 m·s^−2^), and top-speed (km·h^−1^). For the analysis, we used the sum of all GPS metrics of all players that participated in each match, except for the top speed, total distance/minute and total sprint distance/minute that we used the mean of demand of all players that participated by matches.

### 2.6. Technical Analysis

Soccer specific technical performance indicators were measured, and the following match statistics were used: goals scored, goals conceded, total shots, shots on target, assistance, passes, accurate passes, crosses, lost ball, recovered ball, dribbles, and the Instat^®^ index. Data were obtained from Instat^®^ (Instat^®^, Moscow, Russia), a private company which provides teams’ technical performance assessments worldwide. The Instat index^®^ is calculated based on a unique set of key parameters for each playing position (12–14 performance parameters, depending on the position during the game), with a higher numerical value indicating better performance. The exact calculations are trademarked and known only to the manufacturer of the platform [19]. In most general terms, an automatic algorithm considers the player’s contribution to the team’s success, the significance of their actions, opponent’s level, and the level of the competition they play in. The rating is created automatically, and each parameter has a factor which changes depending on the number of actions and events in the match. The weight of the action factors differs depending on the player’s position. The key factors included in the calculation of the Instat index^®^ are position specific and include tackling, aerial duels, set pieces in defense, interceptions for central defenders; number of crosses, number of passes to the penalty area, pressing for full back; playmaking, number of key passes, finishing for central midfielders; pressing, dribbling, finishing, counterattacking for wide midfielders; shooting, finishing, pressing, dribbling for forwards. For the analysis, we used the average of all players in each match.

### 2.7. Statistical Analysis

Data were visually inspected for the existence of outliers (box-plots), tested for normality (Shapiro-Wilk) and homogeneity (Levene), and are presented as means, standard deviation (SD), and 95% confidence intervals (95%CI) of difference between means. All data of perceived exertion and physical and technical parameters for the 2 conditions (2–3 substitutions and 4–5 substitutions) were analyzed with an independent *t*-test. The magnitudes between condition differences were expressed as standardized mean differences and were interpreted using the following thresholds: <0.2, 0.2–0.6, 0.6–1.2, 1.2–2.0, 2.0–4.0, and >4.0, for trivial, small, moderate, large, very large, and near perfect, respectively [20]. The level of significance was set at *p* < 0.05. Statistical analyses were performed using the software package IBM SPSS (V. 25, SPSS Inc., Chicago, IL, USA).

## 3. Results

**Figure 1:**
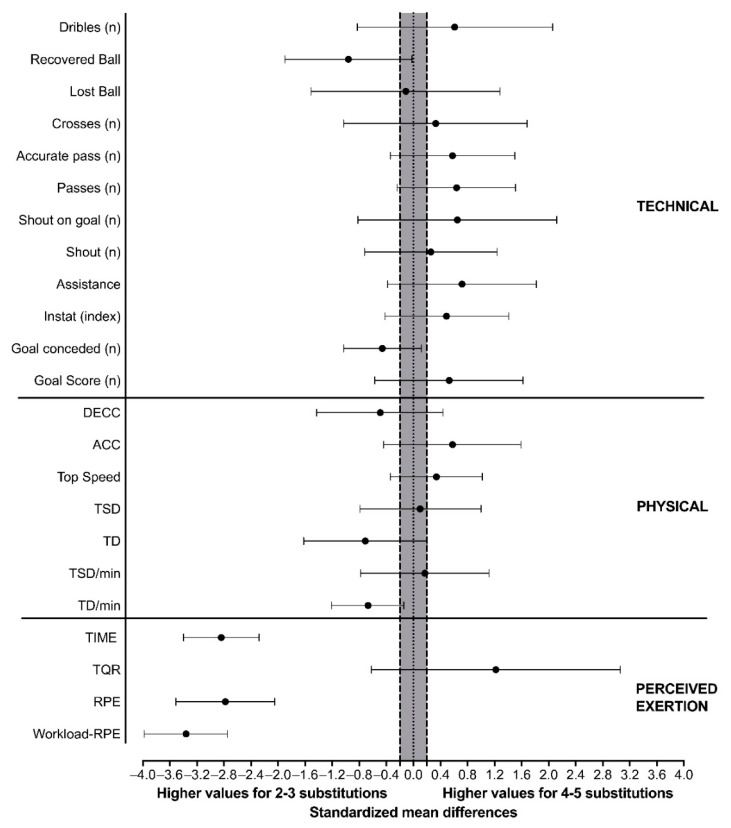
Standardized mean differences for the comparisons of the between substitutions conditions in all parameters of technical, physical, and perceived exertion; The grey area represents the smallest worthwhile difference which corresponds to a small effect size (0.2); Error bars represent the 95% confidence limits.

All matches analyzed were classified as follows: eight matches used two substitutions, 13 matches used three substitutions, two matches used four substitutions, and 15 matches used five substitutions. Then, all matches were grouped into 2–3 (21 matches) and 4–5 substitutions (17 matches). There was no match with no or one substitution. Table 1 displays physical and technical match performance, RPE, workload-RPE, TQR values, and time played of players. The condition of 4–5 substitutions demonstrated significantly lower RPE (*p* ≤ 0.001), workload-RPE (*p* ≤ 0.001), higher TQR (*p* = 0.008), and lower time played by player (*p* ≤ 0.001), compared to 2–3 substitutions. No significant differences were observed in the physical and technical parameters between 4–5 and 2–3 substitutions (*p* > 0.05). Figure 1 shows the standardized mean differences between 2–3 and 4–5 substitutions for all variables.

## 4. Discussion

This is the first study to investigate and compare the effect of the number of substitutions in-match (2–3 vs. 4–5) on physical and technical performance, and psychometrics measured (RPE, workload-RPE, and TQR) in elite women soccer players. The main findings were that 4–5 substitutions demonstrated a lower time played by player, lower RPE and workload-RPE, and higher TQR than 2–3 substitutions. In addition, no difference was observed in any physical or technical parameter of performance between conditions.

Interestingly, the present study demonstrated no differences between both substitution conditions in any physical performance (i.e., GPS metrics). Regardless of the number of substitutions used during the matches, the total metrics of the whole team did not change significantly. We analyzed the sum of metrics from all players, which induced a different perspective from previous studies that compared the relative physical demand (i.e., meters/minutes) among soccer players who played the entire match, were replaced, and substitutes [9,10]. For instance, Bradley et al. [9] demonstrated relative distance covered was inversely related to the time played, i.e., soccer players who covered 112 m/min during the entire match, replaced players who covered 116 m/min, and substitutes who traveled 120 m/min. These findings can be a consequence of fatigue or tactical decisions and adjustments (pacing strategy) in the energetic resources usage by soccer players who play longer periods to enable the completion of the matches [12]. For instance, the players can employ/adjust a pacing strategy that enables the entire match completion, that is, substitute players already know they will be exposed to a shorter match time, which allows them to cover higher distance/min than entire and replaced players [12]. From this perspective, it was conceivable that by increasing the number of substitutions the physical performance of the whole team would also be augmented. However, when we analyzed the sum of absolute values of all players and their mean relative values, using 4 or 5 substitutions did not elicit differences in match running performance compared to 2 and 3 substitutions. It is possible that the amount of distance covered by the team was only slightly affected and the difference did not reach statistical significance. Thus, our findings suggest that the new rule of substitutions may not have an impact on the physical performance of the team during the matches.

Nevertheless, we observed a significant difference in perceived internal load between conditions, according to RPE scale, the 2–3 substitutions were near the “maximal effort” (~8.1) and the 4–5 substitutions were near the “very hard” (~6.7) [18]. These facts can be explained by the fewer minutes played by soccer players during the matches in the 4–5 condition compared to 2–3 (65.5 vs. 76.4 min, respectively). Using five substitutions means that ~50% of the starting team was replaced (excluding the goalkeeper), which enables lower fatigue accumulated by players throughout the matches, mainly at the second half and the end of matches. Accordingly, the workload-RPE was lower in the 4–5 substitutions compared to 2–3 substitutions (441 vs. 623 A.U, respectively). Thus, the new rule of substitution did have a reduction in the perceived exertion of women soccer players. Interestingly, although no difference was observed in the physical performance, results indicate that internal load was reduced. Considering that RPE is affected not only by the intensity but also by the exercise volume [21], this finding seems a consequence of the lower RPE indicated by the substitute and replaced players, who played a shorter time, compared to those that played the entire match. It is reasonable to assume that soccer players who indicate lower RPE have improved wellness and consequently reduced the occurrence of injury and illness, as was reported by previous studies [15,22,23,24].

The shorter time played and lower workload-RPE experienced by substitutes and replaced players are possibly responsible for a superior recovery status after four and five substitutions compared to two and three substitutions. In this regard, higher TQR values suggested a faster recovery after matches. Moreover, Selmi et al. [25] observed correlation of 0.67 between TQR values (before each training session/match) and the successful passes in professional soccer players. Indeed, Kentta and Hassmen [17] indicated that recovery is crucial to avoid maladaptive physical and psychological effects of fatigue. For instance, Brink et al. [16] demonstrated that elite soccer players with higher workload-RPE presented higher injury rates and got sick more frequently. Furthermore, the occurrence of illness seems to be related to worst psychosocial stress and recovery after soccer training and matches. Moreover, a recent meta-analysis demonstrated that a soccer match alters the levels of muscle-injury markers, inflammation, and immunological cell tracking, impairs physical performance, and exacerbates perceptual responses until at least 72 h post-match [13]. Thus, using strategies to improve the balance between stress and recovery status (i.e., increasing the number of substitutions) may improve wellness and mitigate injuries and illnesses in soccer players. These outcomes may help the coaching staff to pre-plan new strategies for training and matches, mainly in the congested period, to improve the recovery for the next match, consequently decreasing the risk of injury and illness.

In line with previous studies [3,8], the technical parameters did not seem to be affected by the physical performance. However, the Instat index of the team was higher (no significant) in 4–5 substitutions compared to 2–3 substitutions (195 vs. 201.3, respectively). The Instat index in soccer is based on wide range of team- and individual-technical statistics. Modric et al. [19] suggested that a higher index demonstrates superior overall technical performance and that the Instat index may be related to the final game outcome (loss, draw, or win). It is conceivable that players with lower fatigue levels during the matches (i.e., substitute players) perform more efficient technical actions than those with higher fatigue (i.e., replaced players or those who played the entire match). For instance, Ferraz et al. [26] showed the negative influence of fatigue upon ball velocity in soccer kicking, which can be related with the impaired neuromuscular performance and may negatively affect coordination skills during the soccer matches. Although we did not observe improved technical performance with higher number of substitutions, more studies are needed to investigate these effects.

Finally, this study is not without limitations. First, it is a case study of only one soccer team. Although we are uncertain if the same findings can be generalized to all soccer teams in all leagues, it provides good evidence that increasing the number of substitutions enhances team recovery and decreases the perception of exertion. Second, this study was restricted to compare 2–3 and 4–5 substitutions because there were no data with no or one substitution during the matches. However, this result provides some evidence on the effect of the number of substitutions on physical and technical performance, perceived exertion, and recovery in women soccer players. Therefore, using four or five substitutions positively affected the balance between stress and recovery from elite women soccer players. Third, although there is a suggestion that injury rate can be influenced by workload, our results indicate lower overall workload in these elite players. Future studies should investigate the effect of the number of substitutions in the incidence of injuries during the matches [24,27]. In summary, the provisory rule of substitutions by FIFA brings a new insight for the future of soccer games.

## 5. Conclusions

The number of substitutions used during the matches did not influence the physical and technical performance of elite women soccer players. However, increasing the number of substitutions decreases the time played and the internal load (i.e., RPE and workload-RPE), which may have positive consequences on the recovery status (i.e., TQR) and the health of athletes by changing the balance between stress and recovery after matches. Therefore, the new provisory rule of substitutions provides some evidence to support the use of more soccer players during the match to improve the balance between stress and recovery.

## Figures and Tables

**Table 1:** Statistics effects between 2–3 and 4–5 substitutions in perceived exertion, physical, and technical variables, mean and SD.

	Variable	2–3 Substitutions	4–5 Substitutions	*p* Value	Mean Difference and 95% CI
Perceived Exertion	RPE (a.u.)	8.1 ± 0.8	6.7 ± 0.7 *	*p* < 0.001	1.4 (0.84–2.00)
	TQR (a.u.)	13.1 ± 0.8	13.9 ± 0.5 *	*p* = 0.008	0.8 (0.2–1.5)
	Time (min)	76.4 ± 4.4	65.5 ± 1.7 *	*p* < 0.001	10.9 (8.6–13.2)
	Workload-RPE (a.u)	623 ± 85	441 ± 53 *	*p* < 0.001	182 (128.7–234.1)
Physical	TD/min (m/min)	106 ± 6	102 ± 6	*p* = 0.064	4 (−0.2–8.1)
	TSD/min (m/min)	7.7 ± 1.2	8.2 ± 1.8	*p* = 0.340	0.5 (−1.5–0.5)
	TD (km)	98.6 ± 5.0	98.0 ± 4.0	*p* = 0.650	0.6 (−2.4–3.8)
	TSD (m)	7002 ± 1191	7465 ± 1614	*p* = 0.316	463 (−1386–460)
	Top Speed (km/h)	26.4 ± 0.6	26.8 ± 0.5	*p* = 0.074	0.4 (−0.8–0.04)
	ACC (n)	715 ± 81	736 ± 69	*p* = 0.404	21 (−71.7–29)
	DECC (n)	913 ± 88	910 ± 67	*p* = 0.909	3 (−50–59)
Technical	Goals conceded (n)	0.38 ± 0.6	0.35 ± 0.6	*p* = 0.886	0.03 (−0.36–0.42)
	Goals scored (n)	2.7 ± 2.2	2.9 ± 1.9	*p* = 0.807	0.2 (−1.5–1.2)
	Assistance (n)	1.8 ± 1.6	1.9 ± 1.5	*p* = 0.9	0.1 (−1.2–1.1)
	Shots (n)	17.9 ± 8	20.8 ± 7	*p* = 0.296	2.9 (−8.6–2.7)
	Shots on goal (n)	7.9± 3.5	9.0 ± 3.1	*p* = 0.367	1.1 (−3.6–1.4)
	Passes (n)	514 ± 94	533 ± 82	*p* = 0.537	19 (−81.7–43.4)
	Crosses (n)	14.0 ± 5.0	16.4 ± 9.0	*p* = 0.372	2.4 (−7.8–3.0)
	Lost balls (n)	75.6 ± 7.0	70.1 ± 9.0	*p* = 0.073	5.5 (−0.5–11.4)
	Recovered balls (n)	58.1 ± 5.0	54.8 ± 6.0	*p* = 0.142	3.3 (−1.1–7.6)
	Dribbles (n)	28.1 ± 9.0	29.2 ± 9.0	*p* = 0.734	1.1 (−7.7–5.5)
	Accurate passes (n)	418 ± 92	441 ± 79	*p* = 0.669	23 (−84–38)
	Instat (index)	195 ± 14.8	201.3 ± 13.4	*p* < 0.001	6 (−1.08–0.21)

Note: TD/min = total distance per minute; TSD/min = total sprint distance per minute; TD = Total distance; TSD = total sprint distance; ACC = acceleration; DECC = deceleration; min = minute; * significant differences between conditions (*p* < 0.05).

## Data Availability

The data presented in this study are available on request from the corresponding author.
